# Supplementary material for: Sound feature representations decorrelate across the mouse auditory pathway
Source: PLoS Biol. 2025 Oct 24;23(10):e3003452. doi: 10.1371/journal.pbio.3003452 (PMC12571308; doi:10.1371/journal.pbio.3003452)
Supplement: S1 Table — Table summarizing the values and statistics of data plotted in Fig 4. For each row, the top value is Mean ± SEM for the region and the bottom value is the Wilcoxon rank-sum test between the region and the previous region (IC against CN, and AC against IC). Identity coding: Pure tones, N = 8−3 sound pairs for 0.3−2.1 octave difference; Complex, N = 105 sound pairs. Intensity coding: Pure tones, N = 14 sound pairs; Complex, N = 15 sound pairs. Significant differences are marked in bold. (DOCX) [file pbio.3003452.s007.docx]

| **Identity coding** | | | | |
| --- | --- | --- | --- | --- |
| **Category** | **ΔOctaves** | **CN** | **IC** | **AC** |
| Pure tones | 0,3 | 0.62±0.05 | 0.57±0.09 | 0.52±0.08 |
|  |  | / | 8,59E-01 | 6,78E-01 |
|  | 0,6 | 0.43±0.03 | 0.46±0.05 | 0.32±0.08 |
|  |  | **/** | 4,01E-01 | 9,29E-02 |
|  | 0,9 | 0.38±0.03 | 0.33±0.05 | 0.22±0.06 |
|  |  | **/** | 3,98E-01 | 1,76E-01 |
|  | 1,2 | 0.35±0.02 | 0.34±0.06 | 0.18±0.04 |
|  |  | **/** | 9,17E-01 | 1,16E-01 |
|  | 1,5 | 0.35±0.04 | 0.35±0.09 | 0.17±0.06 |
|  |  | **/** | 8,93E-01 | 2,25E-01 |
|  | 1,8 | 0.33±0.04 | 0.39±0.07 | 0.11±0.02 |
|  |  | / | 2,73E-01 | 6,79E-02 |
|  | 2,1 | 0.32±0.07 | 0.43±0.15 | 0.11±0.03 |
|  |  | / | 2,85E-01 | 1,09E-01 |
| Complex | / | 0.73±0.01 | 0.67±0.01 | 0.33±0.02 |
|  |  | / | **5,73E-08** | **5,68E-36** |
| **Intensity coding** | | | | |
| **Category** | **/** | **CN** | **IC** | **AC** |
| Pure tones | / | 0.73±0.07 | 0.47±0.1 | 0.54±0.06 |
|  |  | **/** | **3,55E-02** | 5,51E-01 |
| Complex | / | 0.85±0.02 | 0.76±0.03 | 0.31±0.03 |
|  |  | / | **4,09E-02** | **6,55E-04** |
