# Supplementary material for: Sound feature representations decorrelate across the mouse auditory pathway
Source: PLoS Biol. 2025 Oct 24;23(10):e3003452. doi: 10.1371/journal.pbio.3003452 (PMC12571308; doi:10.1371/journal.pbio.3003452)
Supplement: S2 Table — Table summarizing the values and statistics of data plotted in Fig 5. For each row, the top value is Mean ± SEM for the region and the bottom value is the Wilcoxon rank-sum test between the region and the previous region (IC against CN, and AC against IC). Significant differences are marked in bold. Periodic modulations N = 25 sound pairs for each difference; Periodic modulations against pure tones, N = 42 sound pairs; Linear modulations N = 13 sound pairs; Linear modulations against pure tones, N = 13 sound pairs. (DOCX) [file pbio.3003452.s008.docx]

| **Amplitude modulation coding** | | | | |
| --- | --- | --- | --- | --- |
| **Category** | **ΔOctaves** | **CN** | **IC** | **AC** |
| Periodic modulations | 1 | 0.98±0.01 | 0.88±0.02 | 0.79±0.02 |
|  |  | **/** | **1,53E-06** | **2,85E-03** |
|  | 2 | 0.97±0.01 | 0.71±0.03 | 0.59±0.02 |
|  |  | **/** | **1,28E-06** | **9,98E-04** |
|  | 3 | 0.95±0.01 | 0.56±0.05 | 0.47±0.03 |
|  |  | **/** | **2,67E-05** | 8,65E-02 |
|  | 4 | 0.93±0.01 | 0.51±0.08 | 0.33±0.06 |
|  |  | **/** | **7,76E-04** | 1,21E-01 |
|  | 5 | 0.91±0.03 | 0.39±0.12 | 0.25±0.11 |
|  |  | **/** | **1,17E-02** | 5,75E-01 |
| Periodic modulations against pure tones | / | 0.8±0.01 | 0.47±0.03 | 0.46±0.02 |
|  |  | / | **8,42E-08** | **3,41E-02** |
| Linear modulations | Up versus Down | 0.98±0.01 | 0.9±0.04 | 0.85±0.03 |
|  |  | / | **2,09E-02** | 1,55E-01 |
| Linear modulations against pure tones | Similar starting intensity | 0.87±0.04 | 0.74±0.06 | 0.86±0.05 |
|  |  | **/** | **2,62E-02** | 6,30E-02 |
|  | Opposite starting intensity | 0.89±0.03 | 0.81±0.04 | 0.73±0.05 |
|  |  | **/** | **1,42E-02** | 1,70E-01 |
