# Supplementary material for: Sound feature representations decorrelate across the mouse auditory pathway
Source: PLoS Biol. 2025 Oct 24;23(10):e3003452. doi: 10.1371/journal.pbio.3003452 (PMC12571308; doi:10.1371/journal.pbio.3003452)
Supplement: S3 Table — Table summarizing the values and statistics of data plotted in Fig 6. For each row, the top value is Mean ± SEM for the region and the bottom value is the Wilcoxon rank-sum test between the region and the previous region (IC against CN, and AC against IC). Significant differences are marked in bold. Chords N = 195 sound pairs; Chords against pure tones, N = 50 sound pairs; Noise bandwidth, N = 8−3 sound pairs for 0.5−3 octave difference; Noise again. (DOCX) [file pbio.3003452.s009.docx]

| **Multi-frequency coding** | | | | |
| --- | --- | --- | --- | --- |
| **Category** | **/** | **CN** | **IC** | **AC** |
| Chords | / | 0.72±0.01 | 0.77±0.02 | 0.67±0.02 |
|  |  | **/** | **3,76E-10** | **1,90E-09** |
| Chords against pure tones | / | 0.89±0.01 | 0.87±0.01 | 0.74±0.03 |
|  |  | / | 4,79E-01 | **2,38E-05** |
| **Category** | **ΔOctaves** | **CN** | **IC** | **AC** |
| Noise bandwidth | 0,5 | 0.96±0.01 | 0.99±0.0 | 0.91±0.01 |
|  |  | **/** | **1,80E-02** | **1,80E-02** |
|  | 1 | 0.93±0.02 | 0.96±0.01 | 0.85±0.02 |
|  |  | **/** | 1,16E-01 | **2,77E-02** |
|  | 1,5 | 0.89±0.03 | 0.92±0.02 | 0.85±0.03 |
|  |  | **/** | **4,31E-02** | **4,31E-02** |
|  | 2 | 0.85±0.03 | 0.88±0.02 | 0.8±0.03 |
|  |  | / | 4,65E-01 | 6,79E-02 |
|  | 2,5 | 0.84±0.04 | 0.82±0.03 | 0.73±0.03 |
|  |  | / | 1,00E+00 | 1,09E-01 |
|  | 3 | 0.84±0.01 | 0.76±0.06 | 0.73±0.02 |
|  |  | / | 1,80E-01 | 6,55E-01 |
| **Category** | **Frequency content** | **CN** | **IC** | **AC** |
| Noise against pure frequencies | Broad | 0.76±0.02 | 0.55±0.04 | 0.46±0.03 |
|  |  | / | **9,18E-03** | **1,57E-02** |
|  | Ramps | 0.6±0.06 | 0.3±0.08 | 0.28±0.05 |
|  |  | / | **6,91E-03** | 3,86E-01 |
