# Supplementary material for: Sound feature representations decorrelate across the mouse auditory pathway
Source: PLoS Biol. 2025 Oct 24;23(10):e3003452. doi: 10.1371/journal.pbio.3003452 (PMC12571308; doi:10.1371/journal.pbio.3003452)
Supplement: S4 Table — Table summarizing the values and statistics of data plotted in Fig 7. For each row, the top value is Mean ± SEM for the region and the bottom value is the Wilcoxon rank-sum test between the region and the previous region (IC against CN, and AC against IC). Significant differences are marked in bold. Sweep direction N = 5 sound pairs; Sweep against pure tones, N = 10 sound pairs; Complex direction, N = 15 sound pairs. (DOCX) [file pbio.3003452.s010.docx]

| **Frequency modulation coding** | | | | |
| --- | --- | --- | --- | --- |
| **Category** | **/** | **CN** | **IC** | **AC** |
| Chirps direction | / | 0.98±0.01 | 0.91±0.02 | 0.65±0.06 |
|  |  | **/** | **4,31E-02** | **4,31E-02** |
| Chirps against pure tones | / | 0.95±0.01 | 0.68±0.04 | 0.61±0.05 |
|  |  | / | **5,06E-03** | 1,14E-01 |
| Complex direction | / | 0.98±0.0 | 0.96±0.01 | 0.84±0.02 |
|  |  | **/** | **3,56E-02** | **6,55E-04** |
