# Supplementary material for: Sound feature representations decorrelate across the mouse auditory pathway
Source: PLoS Biol. 2025 Oct 24;23(10):e3003452. doi: 10.1371/journal.pbio.3003452 (PMC12571308; doi:10.1371/journal.pbio.3003452)
Supplement: S5 Table — Table summarizing the values and statistics of data plotted in S2 Fig. For each row, the top value is Mean ± SEM for the region and the bottom value is the Wilcoxon rank-sum test between the region and the previous region (IC against CN, and AC against IC). Identity coding: Pure tones, N = 8−3 sound pairs for 0.3−2.1 octave difference; Complex, N = 105 sound pairs. Intensity coding: Pure tones, N = 14 sound pairs; Complex, N = 15 sound pairs. Significant differences are marked in bold. (DOCX) [file pbio.3003452.s011.docx]

| **Identity coding** | | | | |
| --- | --- | --- | --- | --- |
| **Category** | **ΔOctaves** | **CN** | **IC** | **AC** |
| Pure tones | 0,3 | 0,58±0,05 | 0,57±0,07 | 0,48±0,07 |
|  |  | / | 6,78E-01 | 4,41E-01 |
|  | 0,6 | 0,39±0,03 | 0,44±0,04 | 0,3±0,07 |
|  |  | **/** | 2,08E-01 | **3,57E-02** |
|  | 0,9 | 0,34±0,03 | 0,35±0,03 | 0,21±0,05 |
|  |  | **/** | 8,66E-01 | 9,10E-02 |
|  | 1,2 | 0,32±0,02 | 0,37±0,04 | 0,16±0,04 |
|  |  | **/** | 4,63E-01 | **2,77E-02** |
|  | 1,5 | 0,31±0,03 | 0,38±0,05 | 0,14±0,04 |
|  |  | **/** | 2,25E-01 | **4,31E-02** |
|  | 1,8 | 0,28±0,03 | 0,36±0,05 | 0,06±0,02 |
|  |  | / | 6,79E-02 | 6,79E-02 |
|  | 2,1 | 0,27±0,04 | 0,44±0,01 | 0,14±0,03 |
|  |  | / | 1,09E-01 | 1,09E-01 |
| Complex | / | 0,44±0,01 | 0,37±0,01 | 0,32±0,02 |
|  |  | / | **3,92E-23** | **3,36E-06** |
| **Intensity coding** | | | | |
| **Category** | **/** | **CN** | **IC** | **AC** |
| Pure tones | / | 0,67±0,06 | 0,48±0,09 | 0,49±0,06 |
|  |  | **/** | 5,55E-02 | 9,17E-01 |
| Complex | / | 0,73±0,02 | 0,65±0,02 | 0,32±0,03 |
|  |  | **/** | **2,68E-02** | **6,55E-04** |
