# Supplementary material for: Sound feature representations decorrelate across the mouse auditory pathway
Source: PLoS Biol. 2025 Oct 24;23(10):e3003452. doi: 10.1371/journal.pbio.3003452 (PMC12571308; doi:10.1371/journal.pbio.3003452)
Supplement: S6 Table — Table summarizing the values and statistics of data plotted in S3 Fig. For each row, the top value is Mean ± SEM for the region and the bottom value is the Wilcoxon rank-sum test between the region and the previous region (IC against CN, and AC against IC). Significant differences are marked in bold. Periodic modulations N = 25 sound pairs for each difference; Periodic modulations against pure tones, N = 42 sound pairs; Linear modulations N = 13 sound pairs; Linear modulations against pure tones, N = 13 sound pairs. (DOCX) [file pbio.3003452.s012.docx]

| **Amplitude modulation coding** | | | | |
| --- | --- | --- | --- | --- |
| **Category** | **ΔOctaves** | **CN** | **IC** | **AC** |
| Periodic modulations | 1 | 0,81±0,01 | 0,6±0,02 | 0,8±0,02 |
|  |  | / | **2,28E-07** | **1,70E-05** |
|  | 2 | 0,79±0,02 | 0,48±0,02 | 0,59±0,02 |
|  |  | **/** | **9,63E-07** | **1,22E-03** |
|  | 3 | 0,78±0,02 | 0,4±0,03 | 0,47±0,03 |
|  |  | **/** | **1,82E-05** | 1,10E-01 |
|  | 4 | 0,78±0,02 | 0,36±0,04 | 0,31±0,09 |
|  |  | **/** | **5,31E-04** | 2,78E-01 |
|  | 5 | 0,79±0,03 | 0,28±0,06 | 0,19±0,15 |
|  |  | **/** | **1,17E-02** | 8,89E-01 |
| Periodic modulations against pure tones | / | 0,8±0,01 | 0,47±0,03 | 0,46±0,02 |
|  |  | / | **1,77E-08** | 4,20E-01 |
| Linear modulations | Up versus Down | 0,8±0,04 | 0,62±0,07 | 0,71±0,06 |
|  |  | **/** | **2,08E-02** | 2,41E-01 |
| Linear modulations against pure tones | Similar starting intensity | 0,82±0,03 | 0,72±0,05 | 0,83±0,04 |
|  |  | **/** | **4,24E-02** | 7,92E-02 |
|  | Opposite starting intensity | 0,82±0,03 | 0,77±0,03 | 0,64±0,05 |
|  |  | **/** | 1,40E-01 | **3,31E-03** |
