# Supplementary material for: Sound feature representations decorrelate across the mouse auditory pathway
Source: PLoS Biol. 2025 Oct 24;23(10):e3003452. doi: 10.1371/journal.pbio.3003452 (PMC12571308; doi:10.1371/journal.pbio.3003452)
Supplement: S7 Table — Table summarizing the values and statistics of data plotted in S4 Fig. For each row, the top value is Mean ± SEM for the region and the bottom value is the Wilcoxon rank-sum test between the region and the previous region (IC against CN, and AC against IC). Significant differences are marked in bold. Chords N = 195 sound pairs; Chords against pure tones, N = 50 sound pairs; Noise bandwidth, N = 8−3 sound pairs for 0.5−3 octave difference; Noise against pure tones, N = 10 sound pairs for Broad, 8 sound pairs for Ramps. (DOCX) [file pbio.3003452.s013.docx]

| **Multi-frequency coding** | | | | |
| --- | --- | --- | --- | --- |
| **Category** | **/** | **CN** | **IC** | **AC** |
| Chords | / | 0,71±0,01 | 0,77±0,01 | 0,68±0,02 |
|  |  | **/** | **1,69E-09** | **5,03E-08** |
| Chords against pure tones | / | 0,89±0,01 | 0,87±0,01 | 0,74±0,03 |
|  |  | **/** | 1,63E-01 | **3,13E-05** |
| **Category** | **ΔOctaves** | **CN** | **IC** | **AC** |
| Noise bandwidth | 0,5 | 0,91±0,01 | 0,89±0,01 | 0,92±0,01 |
|  |  | / | **4,25E-02** | 1,28E-01 |
|  | 1 | 0,88±0,02 | 0,87±0,01 | 0,85±0,02 |
|  |  | **/** | 7,53E-01 | 3,45E-01 |
|  | 1,5 | 0,84±0,02 | 0,83±0,01 | 0,84±0,02 |
|  |  | **/** | 5,00E-01 | 8,93E-01 |
|  | 2 | 0,81±0,03 | 0,81±0,02 | 0,8±0,02 |
|  |  | / | 1,00E+00 | 7,15E-01 |
|  | 2,5 | 0,8±0,03 | 0,77±0,02 | 0,76±0,03 |
|  |  | / | 5,93E-01 | 2,85E-01 |
|  | 3 | 0,8±0,02 | 0,73±0,03 | 0,73±0,01 |
|  |  | / | 1,80E-01 | 6,55E-01 |
| **Category** | **Frequency content** | **CN** | **IC** | **AC** |
| Noise against pure frequencies | Broad | 0,69±0,02 | 0,5±0,02 | 0,42±0,01 |
|  |  | **/** | **9,82E-04** | **2,58E-02** |
|  | Ramps | 0,55±0,06 | 0,31±0,07 | 0,24±0,05 |
|  |  | / | **5,06E-03** | 1,39E-01 |
