# Supplementary material for: Sound feature representations decorrelate across the mouse auditory pathway
Source: PLoS Biol. 2025 Oct 24;23(10):e3003452. doi: 10.1371/journal.pbio.3003452 (PMC12571308; doi:10.1371/journal.pbio.3003452)
Supplement: S8 Table — Table summarizing the values and statistics of data plotted in S5 and S6 Figs For each row, the top value is Mean ± SEM for the region and the bottom value is the Wilcoxon rank-sum test between the region and the previous region (IC against CN, and AC against IC). Significant differences are marked in bold. Sweep direction N = 5 sound pairs; Sweep against pure tones, N = 10 sound pairs; Complex direction, N = 15 sound pairs; Reconstruction (spatial), N = 4 sound pairs; reconstruction (spatio-temporal), N = 4 sound pairs. (DOCX) [file pbio.3003452.s014.docx]

| **Frequency modulation coding** | | | | |
| --- | --- | --- | --- | --- |
| **Category** | **/** | **CN** | **IC** | **AC** |
| S5: Sweep direction | / | 0,4±0,1 | 0,27±0,08 | 0,31±0,11 |
|  |  | **/** | **4,31E-02** | 6,86E-01 |
| S5: Sweep against pure tones | / | 0,74±0,05 | 0,56±0,07 | 0,45±0,06 |
|  |  | / | **5,06E-03** | 1,14E-01 |
| S5: Complex direction | / | 0,74±0,02 | 0,57±0,02 | 0,78±0,02 |
|  |  | / | **6,55E-04** | **6,55E-04** |
| S6: Reconstruction Sound against Reconstruction Response (Spatial) | / | 0,57±0,01 | 0,41±0,01 | 0,38±0,01 |
|  |  | / | 6,79E-02 | 1,00E+00 |
| S6: Reconstruction Sound against Reconstruction Response (Spatio-temporal) | / | 0.82±0.01 | 0.72±0.01 | 0.51±0.01 |
|  |  | / | 6,79E-02 | 6,79E-02 |
