# Supplementary material for: Sound feature representations decorrelate across the mouse auditory pathway
Source: PLoS Biol. 2025 Oct 24;23(10):e3003452. doi: 10.1371/journal.pbio.3003452 (PMC12571308; doi:10.1371/journal.pbio.3003452)
Supplement: Attachment — Submitted filename: Response to referees.docx [file pbio.3003452.s017.docx]

REBUTTAL LETTER, Gosselin et al.

**Reviewer #1** (Tetsufumi Ito signed his report): The manuscript by Gosselin and colleagues recorded many neurons in the cochlear nucleus (CN), inferior colliculus (IC), and auditory cortex (AC) and analyzed the similarity of responses to various sound stimuli using Pearson's correlation. They concluded that the neuronal population in the CN responded more similarly to various stimuli than higher centers. In the IC and AC, the similarity of population activity decreased, suggesting sparser coding in the higher centers. The degree of reduction in similarity within the hierarchy differed among stimulus types: AM coding mainly occurred in the IC, whereas tuning to harmonic sounds emerged in the AC. The method is clear, and the paper is well-written. I have several comments related to the methodology of the study.

To record population activity, calcium imaging was employed for the AC, whereas multichannel electrodes were used for other regions. Obviously, the temporal resolution of calcium imaging (~150 ms) is inferior to unit recording (on the order of sub-milliseconds), as the authors mentioned, indicating that AC activity reflects time-averaged firing within the temporal resolution window. The authors concluded that this was not an issue because, in their study, feature-specific activity was defined as the average firing rate to the stimuli. However, it should be noted that in lower centers, neurons rely more on temporal coding (i.e., phase-locking to the stimulus time course) rather than rate coding, and this temporal coding is reshaped into rate coding in higher centers. The authors should at least discuss the similarity of temporal coding activity in the lower and higher centers.

>> We fully agree that for temporal acoustic features, the temporal dimension in the code is more important in subcortical regions. Therefore, all figures describing the evolution of feature tuning (Fig. 4-7) had been reproduced with a quantification of the tuning when temporal information is included in the analysis. As expected by the referee, these figures show that temporal information largely improves tuning for temporal features in the periphery. We added a few lines in the discussion to emphasize this aspect:

“Importantly, our analysis focuses on the classical definition of response tuning, based on the time averaged response of the neurons. Adding temporal information improves population tuning, particularly in more peripheral regions. This reflects the fact that all the information necessary to identify acoustic features is already present at the auditory system input, for a large part under the form of temporal activity patterns. As shown previously, information present in the temporal domain progressively transfers to neuron-specific patterns along the auditory system [19]. This transformation is an essential computation of the auditory system which is specifically identified by tuning measures based on time-averaged activity.”

Taking into account the time resolution of the calcium imaging (150 ms), the duration of the stimuli (500 ms) seems slightly too short. Some stimuli even have shorter durations. The authors should provide a justification for employing short-duration stimuli.

>> We agree with the referee that the issue of time resolution is important. We have extensively treated it for similar measurement in our previous paper Bagur et al. 2025, Science Adv, with time-scale specific decoding analysis, showing that for our 500ms sounds the cutoff resolution at which sound specific information can be found is ~3 Hz for 2P and ~30 Hz for electrophysiology (Fig. R1) despite the millisecond resolution of spike time determination.

**Fig. R1.** Bagur et al. 2025 (Figure 4E) (**E**) Left: Sketch illustrating the decomposition of population responses by timescale. Right: Mean decoding accuracy based on successive Fourier coefficients of neural responses. 0 Hz = spatial code. As expected, two-photon data only contained information up to 3 Hz, whereas electrophysiology data were informative even up to 30 Hz.

This figure clearly shows that most sound pairs of the dataset can be decoded with the resolution of calcium imaging ~150 ms (3 Hz). Indeed, the decoding accuracy peaks at 2 Hz resolution for all recording techniques. Moreover we have shown that even for electrophysiology data, information contained in frequency bands higher than 3Hz is redundant to information contained in higher frequency bands (Fig. R2). Therefore, a 3Hz resolution is sufficient to decode all sounds, even if some redundant information is present at faster time scales.

All these quantifications demonstrate that 500 ms sounds are appropriate for this study. This can be also intuitively understood by observing the clear separation between onset and offset responses for these sounds, as seen in sample response traces of Figure 2 and and more precisely detailed in a parallel study published in BioRxiv (Lamothe et al., <https://www.biorxiv.org/content/10.1101/2025.05.19.654889v1>). If onset and onset responses can be well separated, the 500ms duration of the sounds is appropriate for calcium imaging.

Following the referee’s comment we have added a justification of this value in the results:

“In the AC, all regions, but majoritarily primary fields, down to layer 5 were imaged using 2-photon calcium microscopy, and the calcium traces were linearly deconvolved [39], providing a temporal resolution of ~150 ms, which is sufficient to retrieve the temporal information necessary to discriminate 500 ms sounds and a few shorter sounds [19] (Fig. 1F).”

**Fig. R2.** Bagur et al. 2025 (Figure 4F) (**F**) Left: Sketch illustrating the decomposition of population responses by timescale and the concatenation of successive Fourier coefficients to accumulate increasingly fine timescales. Right: Mean decoding accuracy based on cumulative Fourier coefficients of neural responses.

The interval between stimuli is set at 1 second. This is too short for the AC and some neurons in the IC, since higher-order auditory centers have a ~2-second time integration window (Shymkiv et al., 2025), which allows for adaptation to the stimuli. The authors should analyze the degree of adaptation caused by repetitive stimulation.

We are aware that adaptation acts over long-time scales in the auditory cortex, as shown by multiple labs. We have investigated this extensively in a recent publication (Jamali et al., Elife, 2024) showing also a longer than 2s interaction time window. However in the same study we showed that adaptation over this1-2s time scale does not significantly change tuning properties although it may reduce overall responsiveness (Fig. R3) (Jamali et al., Elife, 2024). Since our population tuning measure, based on Pearson correlation, is not sensitive to the overall amplitude of population activity, this reduction will therefore not have an impact on our conclusions. This is why we have chosen to use an interval of 1s.

Fig. R3 (Jamali et al. 2024, Fig. 2*C) (****C****) Responses of all cross-validated neuron clusters to sequences of fives sounds (sounds are four A’s followed by A or B, 250ms between sound onset) for a dataset of auditory cortex neurons imaged with two-photon calcium imaging (neurons numbers are shown on the right column and cluster number on the left). The interval between each sequence is 1.5s, generates adaptation when AAAAB is the repeated sequences. In block in which AAAAA is repeated and AAAAB is rare the time interval between B sounds is too large for adaptation to occur. The responses to B is diminished when AAAAB is repeated, but this does not change tuning.*

The authors found that frequency tuning specificity is already established in the CN and does not improve in the higher hierarchical centers. As they pointed out, this is surprising because tuning sharpness and non-linearity typically increase during hierarchical processing. They should analyze the raw firing properties of individual neurons to identify the factors that make the population's frequency tuning stable across the hierarchy. For other stimuli, it would be more informative to analyze the raw firing activity of each neuron and the relationships between firing activities while preserving the temporal information of the firing activities, which is lacking in the study's analysis.

Following the referee’s suggestion, we measured the half-width of pure tone tuning curves (70 dB tones) in single cells. We observed that the distribution of tuning width is conserved along the hierarchy, corroborating our observations at the population level. These results, shown below, are now integrated in Fig. S2 and quoted in the result section of the manuscript:

“These results emphasize that the fine-scale precision of sound frequency tuning is already established in the cochlear nucleus and is not improved throughout the auditory system. We also observed that the half-width of pure tone tuning curves in single neurons is similar across the cochlear nucleus, inferior colliculus and auditory cortex, with a broad distribution of tuning widths at all stages (**Fig. S2**). This observation corroborates the population tuning measure.”

We have also quoted this result in the discussion, where we more clearly emphasized also that these results are compatible with previous observations.

“Strikingly, however, tuning to frequency for small differences (0.3 octave) does not improve between the cochlear nucleus and the auditory cortex, as seen both at population and single cell levels (**Figs. 4 and S2**), meaning that single frequency information is not further decorrelated, confirming a previous independent observation [[19]](https://www.zotero.org/google-docs/?eracIQ). This result is surprising given the complex evolution of single cell pure tone tuning curves across the auditory system from V-shaped curves in the auditory nerve to a mixture of simple, multipeak and complex tuning curves in the cortex [[36]](https://www.zotero.org/google-docs/?fiAGc3), and the existence of ultrasharp tuning curves in the auditory cortex [[34]](https://www.zotero.org/google-docs/?QHjUPk). Our results do not contradict these observations as, for example, the existence of few cells with ultrasharp pure tone tuning (not directly observable in single cells in our study due to the low resolution of our pure tone sample) can easily be compensated at the population level by a few more broadly tuned neurons.”

**Figure S2F&G** : **The distribution of single-cell frequency tuning is conserved across all regions. F.** Distributions of the halfwidth of pure tone tuning curves (70 dB) for all responsive neurons of each datasets. The distribution profiles are shown as violin plots. **G.** Same as **F.** displayed as overlapping histograms with 0.3 octave bins.

Regarding the referee’s suggestion to add single cell tuning quantifications for all other sounds including temporal information, this has been done in the past by the Furukawa lab, whose work is now cited in the discussion of our manuscript (see reference 80). Also we have analysed in detail temporal information with a population tuning measure in four supplementary figures (Figs. S2-5).

Associating a single cell analysis for each population tuning measurement is outside the scope of this study for multiple reasons:

- the main goal of our study is to introduce the concept of population tuning and provide associated measurements
- Single cell analysis has been extensively performed in the past and the results are already available in the literature.
- As shown for pure tone, single cell analysis provides a broad distribution of tuning width, which can be more difficult to interpret, than the population tuning measure. It is unclear to us from the referee’s comment what would be the new insights gained from this.
- It is a study on its own to try and figure out how to link single cell measures to our population tuning measure.

**Reviewer #2:** This study aims to systematically study how sound feature representations evolve across the mouse auditory pathway from the cochlear nucleus to the inferior colliculus and auditory cortex. They use different recording methods with Neuropixels in the CN and IC and two-photon calcium imaging in the AC, which may introduce some challenges (described below). Importantly, they use a noise-corrected population-level representational similarity analysis instead of relying on single neuron tuning making the analysis more robust to variability. Conceptually, this approach (likely correctly) supposes that downstream regions incorporate population-level inputs. This approach reveals that some features, like single frequency tuning, are already well defined at the CN, while others, such as amplitude modulation frequency, frequency modulation direction, intensity, and complex sound identity, become decorrelated and more specifically represented at later stages in a

feature-dependent manner. Some key findings include that the inferior colliculus is critical for processing AM frequency while the auditory cortex is essential for FM direction and sound identity. These results highlight that auditory features representation across the hierarchy is a mix of gradual and stepwise transformations that enhance the specificity of population-level sound representations. I believe all of my comments can be addressed through textual revisions or relatively modest re-analyses, and I am otherwise supportive of the manuscript's eventual publication.

Some concerns include:

1) Methodological inconsistency across regions. The use of different recording modalities across brain regions, Neuropixels in the cochlear nucleus and inferior colliculus versus two-photon calcium imaging in the auditory cortex introduces potential confounds in comparing representational similarity. In particular, differences in temporal resolution and signal-to-noise characteristics could impact measures of tuning to temporal features such as AM and FM. While the authors' use of a population-level, noise-corrected metric helps mitigate these concerns, matched methodologies across regions would have improved interpretability. This would ideally be addressed with a small number of experiments showing that there are no major differences when using the analytical approaches outlined here (i.e. comparing AC population metrics with calcium imaging versus neuropixels). If that is too difficult, then addressing this in the discussion would also be sufficient. For me, I would want to make sure that these findings are not driven by the chosen recording approach so I would opt for option 1. But it's really up to the authors whether they prefer to pursue additional experimental validation or include a paragraph in the discussion that addresses this concern.

We share with the referee concerns about the sampling methods, which is a complex issue, difficult to fully address. In fact, discrepancies between recording techniques have been little investigated. A study conducted in V1 by the Allen Institute (<https://elifesciences.org/articles/69068>) indicates that visual representations measured with calcium imaging and extracellular electrophysiology can differ. Moreover, before the generalisation of calcium imaging, comparisons between loose patch and multielectrode techniques have started a long standing controversy about the biases introduced by multielectrode extracellular recordings. The main issue raised was that multielectrode approach bias sampling towards high firing rate units because spike sorting is mainly efficient for highly spiking neurons. Within this debate, the Zador lab used a low-bias loose patch recording approach and pointed out that sound responses and spontaneous activity in the auditory cortex are sparse (Hromádka et al. 2008, <https://journals.plos.org/plosbiology/article?id=10.1371/journal.pbio.0060016>). Two-photon calcium imaging retrieves this sparse activity and this is why we have always favored calcium imaging in the cortex. This is important for this study because more decorrelated representations tend to rely on sparse responses.

In Bagur et al. 2025, we have compared multichannel electrodes and two-photon imaging in the inferior colliculus and in the thalamus. In these regions, we found a relatively good match in between representation structures but observed, consistent with the above, slightly larger RSA (Representation Similarity Analysis) correlations value with electrophysiology (Fig. R4). As a follow up of this study, with the same sound set as Bagur et al., we have measured sound responses in AC with Neuropixels and extracellular silicon probes. We found that RSA correlation values were highly dependent on the firing rate of the Neuropixels units. Selecting low firing (< 2Hz) neurons (see Fig. R4), we observed RSA correlation values in the same range as with two photon calcium imaging data. Highly firing neurons (> 2Hz) generate more correlated representations (see Fig. R4). We observed that highly firing neurons are much more prominent in the Neuropixels dataset than in the two-photon calcium imaging dataset, leading to a mismatch between the two datasets in the absence of unit selection. This mismatch is a specificity of the auditory cortex because it is the only structure of the auditory system with a sparse representation.

Our current hypothesis is that the prevalence of highly firing neurons in auditory cortex Neuropixels recordings is due to the oversampling of strongly firing neurons, which are likely interneurons for the most part. By contrast, calcium imaging data, even when using the synapsin promoter for GCAMP expression, are dominated by low firing neurons, likely reflecting predominantly the pyramidal cell population. We are currently working on the verification of this hypothesis, but this is a complex undertaking, far beyond the scope of this study. Therefore, we think that contributing multielectrode electrophysiology data from the auditory cortex is premature. To make the reader aware of this issue we now discuss this point in the revised manuscript:

“The refinement of population tuning also relates to the concept representation sparsening [63]. Loose patch recordings, a technique known to have little sampling biases, show that sound representations in the auditory cortex are based on sparsely responding neurons in the auditory cortex [64]. Response specificity to few sounds leads in single neurons to more specific population patterns and to decorrelation. Sparsely responding neurons, the majority of the neurons in the auditory cortex, also display low on-going firing rates [64,65]. This indicates that recording methods which, like multielectrode electrophysiology, preferentially sample highly firing neurons [66], may underestimate decorrelation. It however remains to identify which cell types underlie decorrelated representations in the auditory cortex and how they are sampled by different recording techniques.”.

***Figure R4****:*

*(dark and light gray lines) Average of the RSA matrix for the spatial (light gray) and spatio-temporal (dark gray) representations of sounds in the mouse inferior colliculus (recorded with two-photon calcium imaging, IC, and silicon probes, ICE), auditory thalamus (recorded with two-photon calcium imaging, TH and silicon probes THE) and auditory cortex (recorded with two-photon calcium imaging, AC) in Bagur et al. 2025. (dark and light gray dots) Average of the RSA matrix for the spatial (light gray) and spatio-temporal (dark gray) representations of sounds recorded with Neuropixels in the mouse auditory cortex in a novel dataset for the same sounds as Bagur et al.. We sorted out low (>2Hz) and high (>2Hz) firing neurons and deep (<-350µm) and superficial (>-350µm) layers.*

2) Interpretation of decorrelated representations. The central framework of 'decorrelation' as a signature of improved feature representation is well-motivated and clearly supported by the data. However, the study stops short of linking these representational changes to functional outcomes such as perception or behavior. Without this, the claim that decorrelation facilitates categorization or recognition remains speculative. An explicit test of whether more decorrelated representations improve behavioral discrimination or downstream decoding would be required to strengthen the interpretation. This is, of course, not needed for this particular manuscript but could be discussed at greater length in the discussion.

We thank the referee for this important suggestion. We have shown in previous studies that a lower correlation of the representations of two sounds predicts faster discrimination learning for thesetwo sounds. For example in Bathellier et al. Neuron 2012 (Fig . 7B, shown below in Fig. R5) we have shown that mice take more time to learn to discriminate between two sounds in a Go/NoGo task when the cortical representations of the two sounds are more correlated. We have also shown in Bagur et al. 2025 that highly correlated optogenetic stimulations of the auditory cortex are more difficult to learn than weakly correlated stimulations. We have also shown in Bathellier et al., PNAS 2013, that in reinforcorment learning models low representation correlations lead to faster learning. We discussed these important functional implications in the revised manuscript:

“The decorrelation of representations has strong functional implications. Computational modeling indicates that decorrelated representations ease their memorization by neural networks [60]. We also showed previously that mice learn to discriminate faster sound pairs whose representations are more decorrelated [19,61].”

*Fig. R5: (A) Schematic of the go/no-go sound discrimination paradigm. (B) Learning curves (fraction correct trials calculated in blocks of 100 trials) for the 3 pairs of sounds tested for discrimination (n = 12 mice per sound pair, mean ± SEM). The inset shows the similarity matrix (Pearson correlation coefficient) between averaged global population vectors measured for the three target sounds (4,734 neurons, 74 populations, 14 mice). This matrix shows that according to global cortical responses sounds S2 and S3 are expected to be perceived more similar to each other than to sound S1. This observation correlates with the fact that discrimination of S2 and S3 leads to much slower learning than discrimination between S1 and S2 or S1 and S3.*

3) Lack of subregion-specific treatment of auditory cortex. A central theme of this manuscript is the hierarchical transformation of sound representations across the auditory pathway. However, the analysis of cortical data treats the AC as a single, unified region, without distinguishing among its known subfields (e.g., A1, AAF, A2, DP). Numerous studies have shown that these subregions differ in their connectivity, temporal dynamics, and selectivity for specific sound features. Some, such as A1 and AAF, are thought to lie earlier in the cortical processing hierarchy, while others, like A2 or DP, are associated with higher-order processing or integrative functions. Collapsing across these subfields obscures potentially important distinctions in how feature representations are transformed and may conflate early cortical representations with those arising later in the hierarchy. If the goal is to map how decorrelation and feature tuning evolve along a sensory hierarchy, then

failing to disaggregate auditory cortical subregions undermines the precision of that conclusion. Even if not all subfields could be sampled densely enough for statistical comparisons, it would be important to acknowledge this limitation more directly, or alternatively use relatively standard approaches to determine where the AC recordings are.

The AC recordings are mainly sampled from A1 and AAF the primary auditory fields, although not exclusively. However, our dataset is too small to address regional differences. It would be extremely interesting to identify further modifications of the representation structure in secondary auditory cortex areas, although we think that it is a study on its own, as secondary areas may also display functional specialization as suggested by studies in the cat (e.g. work by S. Lomber), which would lead to task specific decorrelation of representations. We discuss briefly this point in the revised manuscript:

“It also remains to precisely identify how feature tuning specifically evolves in secondary subfields of the auditory cortex, where they could refine in a task-specific fashion. High density sampling of these regions, much beyond the size of the present dataset, is necessary to solve this question.”

Minor

'We trained mice to stay quietly head-fixed' -> How do you know they are quiet? If you don't have microphonic recordings, please edit this to remove 'quietly'.

done
