# Supplementary material for: Sound feature representations decorrelate across the mouse auditory pathway
Source: PLoS Biol. 2025 Oct 24;23(10):e3003452. doi: 10.1371/journal.pbio.3003452 (PMC12571308; doi:10.1371/journal.pbio.3003452)
Supplement: Attachment — Submitted filename: Response_to_referees_auresp_3.docx [file pbio.3003452.s018.docx]

Reviewer #2: The authors were generally responsive to the reviews. To address my comment about auditory cortical subfields, they state they have insufficient data to make sub-region analyses, which is fine and understandable. I agree that could be the topic of another study. They do not address, however, where their AC recordings are from in the existing manuscript other than this comment in the reviewer response: 'The AC  recordings are mainly sampled from A1 and AAF the primary auditory fields, although not exclusively.' Given the focus of this manuscript, it would appear important to demonstrate this with data in the manuscript itself and not just a comment in the reviewer response. This should be relatively easy given their AC data is collected using 2P imaging and they have the spatial location and tuning of each neuron. Specifically, for each mouse, they can estimate the local tonotopic gradient (plot neural frequency tuning as a function of rostrocaudal location) and
empirically confirm whether that particular mouse/recording is from A1, AAF, or otherwise. I leave it to the editor to decide whether this will be needed for final publication. Otherwise, I support publication of this manuscript.

>> We have addressed the referee’s comment by highlighting in the results that we had imaged most primary auditory cortex (A1 and AAF) and by detailing the localization of imaged neurons (and how they were localized). 20% of the neurons could not be localized, only 10% of localized neurons were outside of (A1+AAF) and >75% of the neurons were in A1. We verified that inclusion of non A1 neurons does not change our conclusions in any way.

Results

we retained […] 4217 neurons from 15 imaging sessions mainly in the primary areas of the auditory cortex AC (see Methods).

Methods

In each animal, the auditory cortex tonotopic map was determined using intrinsic imaging, by measuring the change in 780nm light reflectance over the cranial window, during presentation of pure tones with different frequencies in the lightly anesthetized mice (isoflurane) as described previously [[82]](https://www.zotero.org/google-docs/?pR1jgX). We then identified the main subfields of the right mouse auditory cortex according to [[19,83]](https://www.zotero.org/google-docs/?MSZi9d), using the following definitions A1: most posterior and tonotopic field (low to high frequency gradient, ventro-posterior to dorso-anterior), AAF (Anterior Auditory Field): most anterior tonotopic field (low to high frequency gradient: dorsal to ventral), DP (Dorsal Posterior): non tonotopic, dorsal to A1, SRAF (SupraRhinal Auditory Field): small ventral tonotopic field posterior to AAF (low to high frequency gradient: dorsal to ventral),  VPAF (Ventro Posterior Auditory Field): non-tonopic field posterio-ventral to A1. Using blood vessels as landmarks, we localized the neurons recorded with two-photon calcium imaging in the subfield map. 20% of the neurons could not be localized because of the poor quality of intrinsic signals.  The recorded neurons were mainly in primary auditory fields (A1 and AAF) : A1 2686 neurons, AAF : 303 neurons, DP : 358 neurons, SRAF : 6 neurons, VPAF : 12 neurons, Non-localized : 852 neurons.
